# The effects of the use of customized silicone digital orthoses on pre-ulcerative lesions and plantar pressure during walking in people with diabetic neuropathy: A study protocol for a randomized controlled trial

**DOI:** 10.1016/j.conctc.2023.101247

**Published:** 2023-12-23

**Authors:** Maria L.S. Lucoveis, Mônica Gamba, Erica Queiroz Silva, Letícia A.S. Pinto, Isabel C.N. Sacco

**Affiliations:** a School of Medicine, Physical Therapy, Speech and Occupational Therapy Dept., University of Sao Paulo, Sao Paulo, Brazil; b Nursing School at Federal University of São Paulo, São Paulo, Brazil

**Keywords:** Diabetes mellitus, Diabetic neuropathies, Diabetic foot, Plantar pressure, Digital orthosis

## Abstract

**Background:**

People with diabetes and diabetic peripheral neuropathy (DPN) often develop calluses due to toe misalignment and increased plantar pressure. Untreated, these issues can progress into ulcers, making early intervention crucial. This trial protocol aims to evaluate the efficacy and safety of customized silicone digital orthoses in preventing ulcers, pre-ulcerative lesions, and peak pressure during gait in people with DPN.

**Methods:**

In this superiority randomized controlled parallel trial with single-blind assessment, 60 participants will be allocated to the control group (CG) or the intervention group (IG). The CG will receive specialized nurse-administered foot care, including callus removal, nail care guidance, and self-care education. The IG will receive the same care plus a customized silicone orthosis for toe realignment for 6 months. Assessments will occur at baseline and 3 and 6 months for the primary outcomes (pre-ulcerative lesions and ulcer incidence) and secondary outcomes (pressure distribution, foot function and health, quality of life, safety, and comfort). Two-way ANOVAs (p < .05) will assess group, time, and group by time effects following an intention-to-treat approach.

**Conclusion:**

Although recommended for foot ulcer prevention, custom silicone orthosis adoption remains limited due to the low certainty of evidence. This trial seeks to provide more consistent evidence for the use of toe orthoses in preventing callus and ulcer formation for individuals with DPN.

**Trial registry:**

ClinicalTrials.gov (NCT 05683106) “Effects of Customized Silicone Digital Orthoses in People with Diabetic Neuropathy” (registered on December 20, 2022).

## Highlights

• Toe deformations on an insensitive foot are susceptible to increased loads that may lead to hyperkeratosis.
• Silicone orthoses may protect and/or realign deformed toes to prevent ulcers, amputations and callus formation.
• New studies are needed to increase the level of evidence of customized silicone digital orthoses as a preventive strategy.

## 1 Introduction

The prevalence of diabetes mellitus (DM) continues to rise worldwide. According to the latest data, 537 million people between 20 and 79 years of age are affected by DM, corresponding to 9.8 % of the global population [1]. The new estimate is that 643 million people will be affected by DM by 2030 [1]. Poorly controlled DM, associated with DM progression, leads to multiple long-term complications, including diabetic peripheral neuropathy (DPN), diabetic foot ulcers, amputation, and premature death [[2], [3], [4]] [[2], [3], [4]] [[2], [3], [4]].

DPN affects approximately 50 % of people with DM and is considered a first-degree risk factor for developing foot ulcers [5]. DPN is caused by damage to the somatic fibers, which leads to reduced or absent foot sensitivity, decreased proprioception, muscle loss, and postural instability [6]. Another major finding in people with DPN is motor impairments, such as decreased foot-ankle range of motion [7,8]; atrophy of the intrinsic and extrinsic foot-ankle muscles [[9], [10], [11]]; alteration of the biomechanical properties of connective tissue, such as increased hardness of plantar tissue [12] and increased soft tissue thickness and stiffness [13]; alterations in the lower limbs’ muscle activation [14]; decreased conduction velocity of the tibialis anterior [15]; changes in torque generation strategy from the ankle to the hip [16]; alteration of the plantar arches [17]; decrease or displacement of fat pads from the hallux and toes [18]; forefoot and toe deformities, such as claw, hammer, and mallet toe and hallux valgus [19]; prominences of the metatarsal heads [20]; and changes in foot rollover and gait biomechanics [21].

All of these biomechanical and musculoskeletal alterations cause changes in the plantar pressure distribution during gait [22,23], increasing peak pressures, especially at the forefoot. When associated with the loss of protective sensation, those peak pressure increases could culminate in the formation of hyperkeratosis, which is the most classic manifestation on the feet of people with DPN [24]. Hyperkeratosis confers a 11-fold risk for developing an ulcer [25] and is the second most common cause of ulcer recurrence [26]; thus, prevention and timely treatment are crucial to reduce ulcer incidence and recurrence [27]. Approximately 97 % of foot ulcers develop at the forefoot region, with 58 % of these ulcers developing at the hallux and other toes and 39 % at the metatarsal heads [28]. Thus, as most ulcers occur at the forefoot region [[29], [30], [31]] [[29], [30], [31]] [[29], [30], [31]], strategies for ulcer prevention should consider this region the most critical.

The recommended treatment for hyperkeratosis includes debridement and redistribution of plantar pressure on the metatarsal heads and toes [32]. One of the most common procedures is callus debridement, which can result in a reduction of around 26 % of peak pressure over the treated areas [33]. Although debridement is important to reduce hyperkeratosis, it is a palliative approach; if the cause of hyperkeratosis formation (mechanical stresses) is not dealt with quickly, hyperkeratosis will form again. Therefore, it is necessary to adopt strategies to redistribute plantar pressure over the toes and metatarsal heads to reduce abundant calluses and ulcer formation.

One of the strategies that can be adopted for plantar pressure redistribution is toe realignment through customized silicone digital orthoses, recently recommended by the guidelines of the International Working Group on the Diabetic Foot (IWGDF) [32]. However, studies demonstrating the efficacy of silicone digital orthoses for preventing hyperkeratosis are still very limited and of low scientific quality [32,34]. Only one randomized controlled trial has demonstrated that the use of custom orthoses on the forefoot of people with DPN and a high risk of ulceration is safe and reduced the incidence of new pre-ulcer lesions over a 3-month period [35]. In addition, a prospective study indicated that customized silicone digital orthoses were effective for reducing 10 % of peak pressure during gait in people with toe deformities after 18 months, with 82 % of the participants reporting improvements in their gait pattern [36]. There is also some evidence of the effectiveness of this approach in the short term in people with calluses from different etiologies. Leather, gel, and silicone orthoses were shown to acutely reduce 40–50 % of the plantar load at the second toe apex during gait in people with calluses associated with claw or hammer toes [37]. Additionally, customized silicone orthoses alone acutely reduced 30 % of the peak pressure under the toes, but the association of a debridement procedure with customized silicone orthoses reduced the plantar pressure by 54 % in individuals with DPN and calluses in the distal phalanges of the toes [38].

Given this context, silicone digital orthoses, which are non-invasive, customizable using molds made by professionals with prior training, inexpensive, and easy to handle for the users, may be a promising alternative to reduce the risk of ulcerations on the feet of individuals with DPN associated with motor deformities by reducing hyperkeratosis [39]. Thus, the objective of this trial is to evaluate the efficacy and safety of using customized silicone digital orthoses for 6 months compared to the usual recommended podiatric care [32] for the prevention of ulcers and pre-ulcerative lesions at the forefoot and the reduction of peak pressures during gait in people with DPN [32].

## 2 Methods

### 2.1 Trial design and setting

**Fig. 1:**
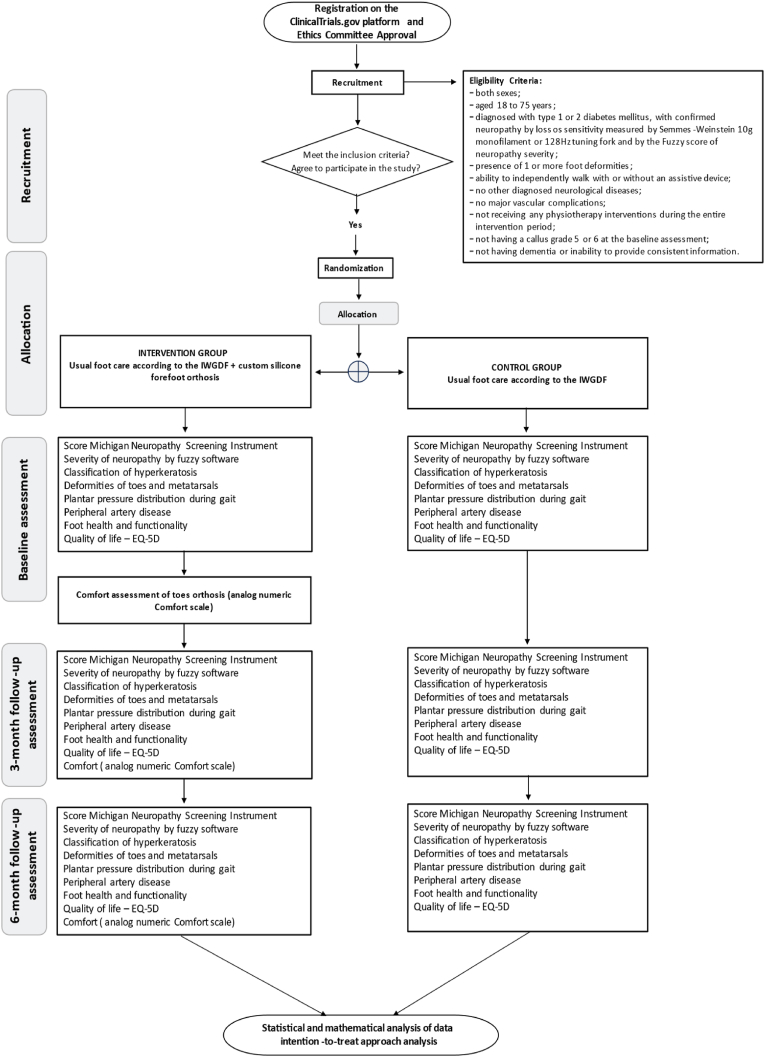
**-** Flowchart showing the stages of the clinical trial.

This will be a superiority controlled clinical parallel trial with random allocation and single-blind assessment. The trial has been approved by the Research Ethics Committee of the School of Medicine at USP (CAAE 65204622.4.0000.0068, 11/24/2022) and registered with ClinicalTrials.gov (NCT05683106, 12/20/2022). Any important protocol modifications will be reported to the relevant parties (funding sources and trial registration). This trial will have an allocation ratio of 1:1 and will follow all SPIRIT recommendations [40]. Fig. 1 presents a flowchart with the steps for the clinical trial.

The trial will be conducted with individuals who have DPN and forefoot deformities (IWGDF risk categories 2 and 3). The participants will be allocated randomly to either the control group(CG), which will receive guidance on preventive foot care and callus debridement, or to the intervention group(IG), which will receive guidance on preventive foot care, callus debridement, and customized silicone digital orthoses according to the forefoot deformity. The CG and IG will receive guidance regarding the use of therapeutic insoles and footwear according to individual needs. IG participants will be evaluated at baseline, 1 week after baseline to inquire about the safety and comfort of the orthoses, and at 3 and 6 months for follow-up measurements. CG participants will be evaluated at baseline and 3 and 6 months after allocation. The assessments will be carried out at a nursing clinic specialized in stomatherapy, where the main researcher performs clinical activities focusing on individuals with DPN.

### 2.2 Participants and recruitment

Participants will be recruited from private clinics linked to the Brazilian Unified Health System (Sistema Unico de Saúde; SUS) that are specialized in preventing and treating the diabetic foot as well as from clinics specialized in treating wounds. Participants will undergo an initial assessment for screening and confirmation of eligibility criteria. Inclusion criteria will be people of both sexes ages 18–75 years who are diagnosed with type 1 or 2 DM with DPN as confirmed by loss of sensitivity measured by Semmes–Weinstein 10-g monofilament, a 128-Hz tuning fork, and the fuzzy score of DPN severity (www.usp.br/labimph/fuzzy); have one or more forefoot flexible deformities (claw, hammer, mallet, or overlapping toes; bunion; prominences of the metatarsal heads; or decrease/displacement of adipose cushion); and are able to independently walk with or without an assistive device. The non-inclusion criteria will be other diagnosed neurological diseases, major vascular complications, receiving any physiotherapy interventions for the lower limbs during the entire intervention period, not performing foot-ankle exercises, having a callus grade 5 or 6 at baseline, major amputations in both limbs (above the ankle), minor amputations in the foot that will receive the orthosis (Chopart, Lisfranc, transmetatarsal), and the presence of dementia or inability to provide consistent information.

### 2.3 Randomization, allocation, and blinding

Participants will be randomly allocated to the IG or CG by a random numeric sequence prepared by the WINPEPI software (PEPI-for-Windows) [41] by an independent researcher who will be unaware of the numeric codes for the groups. This sequence will be generated into 16 blocks of four to eight people per block. The numeric sequence will be kept in sequentially numbered opaque envelopes according to the order generated by the software. This sequence will be kept confidential and stored in a location that blind assessors will not have access to. Participants will also be stratified according to the risk classification of ulcers (IWGDF grade 2 and 3 equally distributed). Thus, 30 participants will be stratified to the subgroup with ulcer risk 2, and 30 participants will be stratified to the subgroup with ulcer risk 3, totaling 60 participants.

After agreeing to participate in the study and signing an informed consent form, allocation to the groups will be made by another independent researcher who will not know the numeric code identifying the groups. All personal data will be kept confidential before, during, and after the study by coding the participants’ names. Only the nurse and the person receiving the treatment will know the meaning of the code. After allocation to the IG, digital orthoses will be made, and the participant will be able to pick them up 24 h later.

Due to the characteristics of the intervention, it will not be possible for the nurse responsible for making the orthoses to be blinded. Participants also cannot be masked regarding the intervention since the therapy modality to be applied (toe orthoses) is known to the general public, and the differences between intervention and non-intervention are completely evident. However, participants will not be aware of the study's hypothesis.

The data tabulation of all assessments and statistical analyses will be done by an independent researcher who is blinded to the intervention modality and numerical codes identifying the groups. Before each assessment, all participants will be instructed to not reveal which group they belong to (IG or CG), and this procedure will be reiterated at each new re-assessment.

### 2.4 Trial arms

Both the IG and CG participants will be instructed to maintain self-care strategies at home, which include proper cleaning, drying the feet and between the toes, moisturizing the skin, and adequate nail trimming. In case of any participant from either group presenting any foot complications, such as a foot ulcer, the participant should immediately notify the researchers and receive guidance from the research team on how to proceed.

#### 2.4.1 Control group

After randomization, CG participants will be attended by a nurse specializing in stomatherapy, dermatology, or clinical podiatry. Participants will receive foot care for the removal of calluses; referral (if necessary) to the Specialized Rehabilitation Center (Centro Especializado de Reabilitação; CER), a health facility linked to SUS, for the acquisition of insoles and/or custom-made shoes (when indicated); and guidance on the use of therapeutic footwear. After study completion, toe orthoses will be offered to CG participants according to their needs.

#### 2.4.2 Intervention group

IG participants will receive the same instructions as the CG participants in addition to customized silicone orthoses for toe realignment according to their needs. When not using therapeutic footwear, common footwear will be accepted if it provides adequate fit to accommodate the orthoses comfortably in order to avoid possible trauma resulting from improperly sized footwear.

During the reassessments, IG participants will have their orthoses examined and repaired or replaced if necessary. The criteria for discontinuing orthosis use during the study will include moderate to severe pain or any other condition that exposes the participant to discomfort, trauma caused by the use of the orthoses, allergy to the orthosis material, difficulty adapting to the orthoses, and imbalance. Participants will be instructed to promptly report any type of adverse events or discomfort. The criterion for discontinuing the intervention will also include the participant's own withdrawal from the study. If the IG participant stops using the orthoses without any explanation, their participation in the study will be terminated and they will be considered a drop out.

**Fig. 2:**
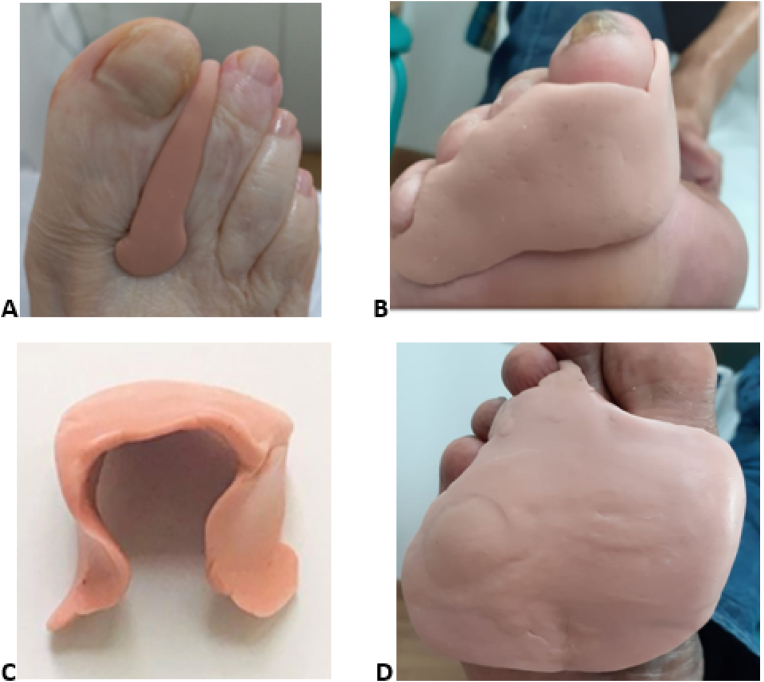
**–** Toe orthosis types: (A) toe separator-type orthosis, (B) subphalangeal orthosis for flexible claw toe, (C) omega-type orthosis, (D) orthosis for protection of the metatarsal heads.

A bunion (or hallux valgus) will be treated with a toe separator type of orthosis to realign the toe (Fig. 2A). For flexible claw toes, a subphalangeal orthosis will be provided for realignment (Fig. 2B). A flexible hammer toe will be treated with an omega-type orthosis that protects the back of the toe without covering the nail, anchoring the support points in the plantar region of the toes (Fig. 2C). Prominences of the metatarsal heads will be treated with a semi-rigid silicone metatarsal head protector orthotic, and in the areas where impact needs to be cushioned, extra soft silicone will be used (Fig. 2D).

To prevent adverse effects, IG participants will received orthosis guidance, such as cleaning the orthoses and interdigital spaces with soap and water, adequately drying the toes and interdigital region, using talcum powder to absorb moisture between the toes, always using the orthoses with appropriate footwear and with socks to promote sweat absorption and prevent orthosis displacement, using the orthoses during all day, except during bath and when sleeping, and monitoring the integrity of the orthoses and always reporting any changes in the feet or orthoses.

Throughout the study, possible adverse effects will be evaluated, such as skin irritation (redness, itching, contact dermatitis, or erosions), discomfort or pain sensation (bulky orthoses), muscle contractions, hyperkeratosis, increased foot sweating, or trauma from friction (caused by an orthosis that has folded or is poorly adjusted to the toes). Adherence to the intervention will be evaluated through scheduled reassessments every 3 months and weekly via WhatsApp messages.

### 2.5 Outcome assessments and timeline

Two researchers blinded to group allocation will perform the baseline and all follow-up assessments. IG participants will be assessed at baseline, after 7 days (via a telephone questionnaire about safety and comfort), at 3 months, and at 6 months. CG participants will be assessed at baseline and 3 and 6 months. Intervals of 3 and 6 months were established for the reassessments following the IWGDF guidelines, which recommend that individuals with DM classified as risk 2 need to be evaluated every 3–6 months and with risk 3 every 1–3 months. Table 1 presents the primary and secondary outcomes and the study period in which they will be evaluated according to the SPIRIT guidelines [40].

**Table 1:** Primary and secondary outcomes of the study following the SPIRIT guidelines.

Outcomes		When participants will be assessed			
Primary	Measures	Baseline	7 days	3 m	6 m
Pre-ulcerative lesions and ulcers	Assessed according Colagiuri et al. (1995), which classifies hyperkeratosis into 6 grades.	**X**		**X**	**X**
**Secondary**	**Measures**				
Plantar pressure distribution during gait	Pressure peak, pressure-time integral, contact area by anatomical masking	**X**		**X**	**X**
Range of motion (ROM) of metatarsofalgeal joint	To assess ROM, a manual goniometer will be used to assess joint amplitude passively and actively	**X**		**X**	**X**
Toes alignment and flexibility	To measure toes alignment, toes will be photographed with markings on the metatarsophalangeal and interphalangeal joints.	**X**			**X**
Foot health and functionality	FHSQ-BR Domain scores	**X**		**X**	**X**
Quality of life	EQ-5D questionnaire	**X**		**X**	**X**
Satisfaction and safety	Satisfaction and Safety Questionnaires		**X**	**X**	**X**
Comfort	Analog numerical comfort scale		**X**	**X**	**X**

**Fig. 3:**
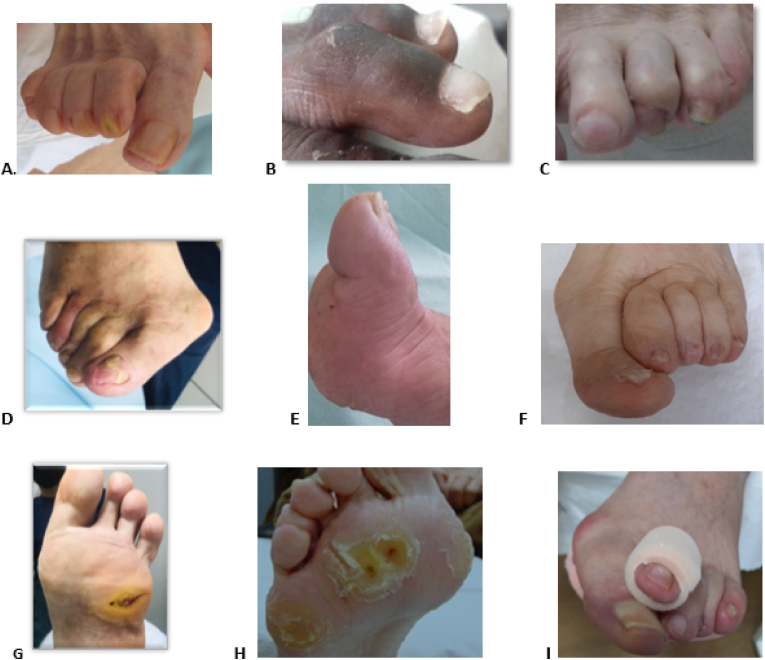
**-** Illustration of deformities at the forefoot region: (A) claw toe, (B) hammer toe, (C) mallet toe, (D) hallux valgus, (E) rigid or reduced hallux, (F) overlapping mobility toes, (G) fat pad atrophy, (H) prominence of the metatarsal heads, (I) supraduct toe.

To ensure the eligibility criteria, all participants will undergo a baseline anamnesis, which will include questions about sociodemographic and anthropometric data, comorbidities, and the risk of developing ulcers. To verify the presence of DPN, peripheral arterial disease, and toe and forefoot deformities, we will follow the recommendations of the International Consensus on the Diabetic Foot [27]. The Semmes–Weinstein 10-g monofilament and a 128-Hz tuning fork will be used to check for loss of tactile and vibratory sensitivity, respectively. Peripheral arterial disease will be verified by palpation of the pedal and posterior tibial pulses in both limbs. The presence of at least one palpable pulse in each limb will rule out peripheral arterial disease. Toe and forefoot deformities, such as claw, hammer, and mallet toes; hallux valgus; reduced mobility; atrophy or displacement of the plantar fat pad; metatarsal head prominences; overlapping toes; and supraducts (Fig. 3), will be identified through observation. Study participants with an ulceration risk level of 2 (with toes deformities and DPN) or 3 according to the guidelines of the IWGDF [27] will be included. Those classified as having a risk of 0 or 1 will not be included in the study.

### 2.6 Primary and secondary outcomes

The incidence of ulcers and pre-ulcerative lesions will be considered the primary outcomes. The plantar pressure distribution during walking, metatarsophalangeal joint range of motion (ROM), toe alignment, foot function, overall foot health, quality of life, satisfaction, safety, and comfort will be secondary outcomes.

#### 2.6.1 Pre-ulcerative lesions and ulcers

**Fig. 4:**
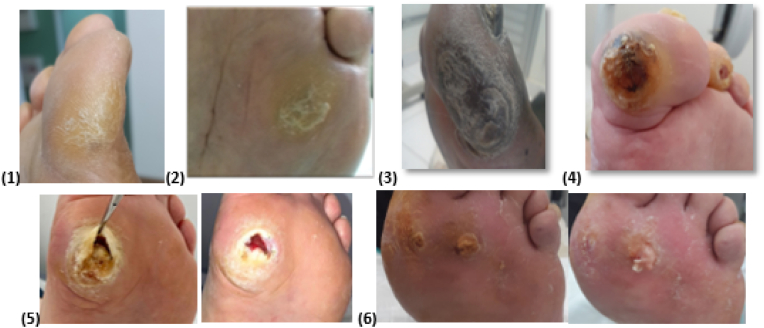
Grades 1 to 6 of severity of pre-ulcerative lesions and ulcers.

The assessment of pre-ulcerative lesions (hyperkeratosis) and ulcers will be performed according to the study by Colagiuri et al. [42], in which pre-ulcerative lesions and ulcers were evaluated using six grades, as follows (Fig. 4):

1. Grade 1: minimal thickening of the keratin layer
2. Grade 2: moderate thickening of the keratin layer
3. Grade 3: marked thickening of the keratin layer
4. Grade 4: callus with subcutaneous bleeding
5. Grade 5: callus with ulcer
6. Grade 6: callus with infected ulcer

Grades 1 to 4 refer to pre-ulcerative lesions, while Grades 5 and 6 are already established ulcers. If the participant is classified as Grade 5 or 6 at baseline assessment, they will not be included in the study but may be included later after the ulcer has healed.

#### 2.6.2 Plantar pressure distribution during gait

A pressure platform (emed-q100; novel, Munich, Germany) will be used to assess pressure distribution during walking. Data will be collected at 100 Hz. Patients will walk through a 5 m walkway in a self-selected and comfortable speed and the platform will be placed close to the end of the walkway, allowing patients to perform 2–3 steps before stepping onto the plate and 1–2 steps after the plate. Immediately before data collection, participants will be instructed to carry out a habituation period to feel comfortable to the environment and to the pressure plate. The walking speed will be recorded (chronometer) and the same speed will be kept in all follow up assessments. At T3 and T6, IG participants will be assessed with socks with and without orthoses. The procedure will be repeated up to three steps on each side. For analysis of the regions of interest, a geometric mask that divides the foot into seven regions—rearfoot, midfoot, medial forefoot, central forefoot, lateral forefoot, toes, and hallux—will be used [43]. Peak pressure, contact area, and pressure–time integral in each sub-plantar area will be analyzed.

#### 2.6.3 ROM of the metatarsophalangeal joint and toe alignment and flexibility

To assess ROM, a manual goniometer will be used to passively and actively measure joint amplitude [44]. To measure toe alignment, toes will be photographed with markings on the metatarsophalangeal and interphalangeal joints at baseline and 6 months, and the alignment will be qualitatively evaluated.

#### 2.6.4 Satisfaction and safety

To evaluate the safety and satisfaction with the use of orthoses, a structured closed questionnaire will be applied consisting of 11 questions related to safety and seven related to satisfaction. Responses will be assessed using a 5-point Likert scale (1 = completely disagree; 2 = disagree; 3 = neither agree nor disagree; 4 = agree; 5 = completely agree). The questionnaire will be administered by phone one week after baseline and repeated at 3 and 6 months from baseline.

#### 2.6.5 Comfort

To evaluate comfort when using the silicone orthoses, a visual numeric scale (where 0 corresponds to very uncomfortable and 10 corresponds to very comfortable when using the orthoses) will be applied.

#### 2.6.6 Foot health and functionality

The Brazilian version of the Foot-Health Status Questionnaire [45] will be used. This instrument is divided into seven domains (pain, function, footwear, general health, physical activity, social capacity, and vitality) receiving a score of 0–100, where 100 represents the best condition and 0 represents the worst. The data will be tabulated in the Foot-Health Status Questionnaire software version 1.03.

#### 2.6.7 Quality of life

The participants will fill out the Euro Quality of Life Instrument-5D (EQ-5D) questionnaire, which is a generic instrument for measuring health-related quality of life and allows for generating an index representing the value of an individual's health state [46]. The EQ-5D algorithm generates a value between −0.59 and 1.00, which represents the possible health status of a person (where 1 represents a perfect health value).

### 2.7 Sample calculation and statistical analyses

The sample size calculation was performed using GPower v. 3.1 software [47] based on the primary outcome variable of the study: the presence of hyperkeratosis (pre-ulcerative lesions). For this outcome, the relative risk (RR) was calculated based on the incidence of hyperkeratosis after treatment with silicone digital orthoses in people with DPN from a previous study [35] using a contingency table. The sample size calculation used the following parameters: RR = 0.4913, test power = 0.80, α = 0.05, and a chi-squared test for contingency tables, resulting in a sample size of 54 participants. Assuming a 10 % loss to follow-up, 60 participants will be needed.

The inferential statistical analysis will be done by adopting an intention-to-treat approach. The imputation of missing values for the analyzed variables will be depend on the nature of the losses. After confirmation of normality (Kolmogorov–Smirnov test), homoscedasticity (Levene test), and imputation of means for missing data of variables with normal distribution, a two-way repeated measures ANOVA will be performed followed by Newman–Keuls post-hoc test to obtain group (CG and IG), time (baseline and 3 and 6 months), and group by time interaction effects. Significant differences will be considered with an alpha of 5 %. For the description of the intervention effect, the effect size (Cohen's d coefficient) and the difference between means with 95 % confidence intervals will be calculated.

### 2.8 Data management

The study steering committee is comprised of one undergraduate student (responsible for blind assessment, data tabulation, and codification), one senior researcher (responsible for data acquisition and monitoring data tabulation), one coordinator (responsible for managing the project), and one assistant researcher (responsible for the recruitment and scheduling of collections). All information collected during the protocol will be entered into an electronic form by those responsible for data collection. All information will be collected on physical and electronic forms by those responsible for data acquisition who are blinded to the participants’ allocation. The integrity and validity of the data will be verified at the time of data entry (edit checks). Identification of potential recruits will be done by the project manager and the research assistant. The research assistant will be trained on how to approach the eligible subjects during the initial recruitment contact for the survey (made by phone calls) and how and when to contact them for follow-up and data collection. After study completion, anonymized data will be published in the public USP repository (https://repositorio.usp.br/).

### 2.9 Data supervision and monitoring

The data monitoring committee (steering committee) and the Faculdade de Medicina da Universidade de São Paulo Board will regularly monitor (depending on the recruitment numbers and collections performed) the study datasets and make recommendations on necessary protocol modifications or termination of all or part of the study. A trimester meeting will be held to facilitate the study's development. All team members can request meetings as needed. All adverse events occurring during the clinical trial period will be recorded. The patients will be advised to report any discomfort and foot pre-ulcerative signs (e.g., blisters and calluses) or foot ulcers to the main researcher, who will ask for the blinded podiatrist nurse to assist the patient.

## 3 Discussion

This paper describes a randomized trial protocol that aims to evaluate the efficacy and safety of customized silicone digital orthoses in preventing ulcers, pre-ulcerative lesions, and peak pressures during gait in people with DPN and risk category 2 or 3. This study is supported by evidence that people with DPN often develop calluses due to toe misalignment and increased plantar pressure. If toe deformities and calluses are not addressed in a timely manner, they can evolve into plantar ulcerations [25,33,48]. Once installed, these ulcerations are difficult to manage and have the potential for amputation. Therefore, early prevention and treatment are crucial.

Conservative treatment for dealing with calluses, including debridement, is the first clinical choice; however, when that fails, surgical treatment is the alternative [49]. Although conservative treatment provides quick relief for patients, it is of limited benefit because if plantar pressure at the forefoot is not redistributed effectively, the callus will soon form again as a way of protecting against chronic excessive mechanical stimulation [50]. Therefore, callus removal must be accompanied by strategies for redistributing plantar pressure. Customized silicone orthoses are a strategy for toe realignment and, consequently, plantar pressure redistribution. A single clinical trial has indicated the efficacy and safety of using customized orthoses at the forefoot to reduce the incidence of pre-ulceration lesions in people with DPN [35]. In addition, other studies have evaluated the use of silicone orthoses aimed at reducing peak pressure with promising results [[36], [37], [38]]. Although the use of silicone orthoses is recommended by the IWGDF guidelines [32], its use worldwide is limited due to a lack of evidence. Thus, a randomized controlled trial addressing the effectiveness of this approach to prevent ulcerations and pre-ulcerative lesions would provide more consistent evidence for the adoption (or not) of this approach as a preventive strategy for people with DPN.

### 3.1 Trial status

The overall status of the trial is currently recruiting patients for the study.

## Author contribution

MLSL and ICNS were responsible for the conception and design of the study; MLSL, EQS, LASP, MG and ICNS are responsible for data acquisition and data analysis; MLSL, EQS, LASP, MG and ICNS for data interpretation and for drafting the paper. All authors read, provided feedback and approved the submitted version.

## Funding

This research is supported by 10.13039/501100002322the Coordenação de Aperfeiçoamento de Pessoal de Nível Superior (Coordination for the Improvement of Higher Education Personnel, CAPES) financial CODE 001. Sacco is a fellow level 1B of the National Council for Scientific and Technological Development (CNPq), Brazil (Process: 302558/2022-5) and Pinto holds a scholarship from CNPq (PIBIC). The funders do not have any role in the study and do not have any authority over any study activity or in the decision to submit the report for publication.

## Declaration of competing interest

We, the authors, would like to submit the paper “The effects of the use of customized silicone digital orthoses on pre-ulcerative lesions and plantar pressure during walking in people with diabetic neuropathy: a study protocol of a randomized controlled trial”, as a study protocol, to the *Contemporary Clinical Trials Communications* for possible future publication. We would like to inform that the present manuscript or its parts have not been published in another journal, is not presently under consideration by another journal, and will not be submitted to another journal before a final editorial decision from the *Contemporary Clinical Trials Communications* is rendered.

One author have been funded by CNPQ and one by CAPES. The paper was written with no influence from the funding agency and no author (will) receive(d) anything of value from the commercial product included in this paper. We also state that we do not keep any commercial relationships that may lead to a conflict of interests.

All the authors were fully involved in the study and preparation of the manuscript, they are in an agreement about what has been written and they are aware of every statement of the conflict-of-interest letter. Each one of the authors has read and concurs with the content in the final manuscript and are aware of every statement of this letter.

## Data Availability

No data was used for the research described in the article.
